# Acquired reactive perforating collagenosis in a patient with multiple comorbidities: a case report from Palestine

**DOI:** 10.3389/fmed.2026.1870746

**Published:** 2026-06-26

**Authors:** Majd Mohsen, Eman Jaber, Qais Naserallah, Montaser Badran, Sameer Mtour, Sami Bannoura, Rabee Adwan

**Affiliations:** 1 Faculty of Medicine, Al-Quds University, Jerusalem, Palestine; 2 Department of Internal Medicine, Al-Maqassed Islamic Charitable Society Hospital, Jerusalem, Palestine; 3 Department of Pathology, Al-Makassed Islamic Charitable Society Hospital, Jerusalem, Palestine; 4 Department of Infectious Diseases, Al-Makassed Islamic Charitable Society Hospital, Jerusalem, Palestine

**Keywords:** acquired reactive perforating collagenosis, chronic kidney disease, diabetes mellitus, heart failure, hyperparathyroidism, Palestine, perforating dermatoses

## Abstract

**Introduction:**

Perforating dermatoses comprise a heterogeneous group of skin disorders characterized by transepidermal elimination of dermal components such as collagen, elastic fibers, or fibrin, resulting in pruritic papulonodular lesions. Four established subtypes are identified: reactive perforating collagenosis (RPC), elastosis perforans serpiginosa, perforating folliculitis, and Kyrle disease. RPC can be hereditary, manifesting in childhood, or acquired (ARPC), which generally develops in maturity and is closely associated with systemic diseases, particularly chronic renal failure and diabetes mellitus.

**Case presentation:**

A 55-year-old male who presented with symptoms of decompensated heart failure, type-2 diabetes, chronic renal failure, and hyperparathyroidism. During hospitalization, he complained of cutaneous pruritus and skin lesions all over his body. Physical examination revealed papules of varying sizes; some progressed into umbilicated nodules featuring central keratotic plugs. Histopathology revealed cup-shaped epidermal invaginations with necrotic debris and transepidermal extrusion of modified collagen, as confirmed by Masson's trichrome stain (MTS). Therefore, a diagnosis of ARPC was established for the first time.

**Intervention and outcome:**

The patient was treated with the Dermovate scalp application, S.C. omalizumab administered monthly, fexofenadine hydrochloride, and body moisturizers. Besides that, treatment for glucose control, fluid overload, and recovery of renal function was also applied. Following up after 1 month revealed minimal improvement in pruritus; however, there was no change in skin lesion status.

**Conclusion:**

Acquired reactive perforating collagenosis remains a rare dermatologic disease. To the best of our knowledge, this represents the first reported case from Palestine. This case highlights the potential coexistence of ARPC with various systemic comorbidities, underscoring the need for comprehensive clinical assessment and exploration of underlying diseases in affected individuals. Furthermore, it highlights the importance of increased clinical awareness, particularly among physicians in similar settings, to consider ARPC in the differential diagnosis of pruritic cutaneous eruptions, especially in patients with associated comorbidities such as diabetes mellitus and chronic kidney disease.

## Introduction

Reactive perforating collagenosis (RPC), first described by Mehregan et al. (1) in 1967, is a subcategory of perforating dermatosis disorders—a rare dermatologic disease marked by the transepidermal passage of altered collagen bundles (2). It is subdivided into two categories: inherited RPC (IRPC), which is the infant and childhood form, and acquired RPC (APRC), which occurs in adults and is frequently associated with systemic disorders, most commonly diabetes mellitus (DM), cardiovascular diseases, hypertension, and chronic kidney disease (CKD), with additional cases linked to malignancy, skin trauma, infections, and drug usage (2, 3).

Since its initial description in 1967, fewer than 50 cases have been documented in the literature (4). Published reports indicate a higher prevalence in males, with a mean age of 60.8 years (±14.40) at presentation (2, 5). The skin lesions are typically multiple pruritic nodules, overlaid by a central adherent keratotic plug. Some may resolve spontaneously, with remaining regions of atrophic scarring and transient hypochromic skin areas (2, 3). It presents frequently on the extensor surfaces of the lower limbs, followed by the trunk and the extensor surface of the upper limbs. However, it rarely involves the face or neck (2).

Trauma, such as scratching, is one of the proposed triggers of transepidermal elimination of altered collagen, leading to ARPC. As a result, the presence of the Koebner phenomenon is an important clinical feature, as new umbilicated papules often develop along scratch marks or areas of friction (2). In a case series by Kollipara et al. (6) it was observed in 40% of cases.

The definitive diagnosis of ARPC must be based on clinical assessment and supported by histopathological evidence, in accordance with the diagnostic criteria proposed by Faver et al. (3). Treatment of ARPC focuses on symptomatic relief and providing adequate control of the associated systemic diseases (2).

Here, we report a case of ARPC in a 55-year-old male with multiple systemic comorbidities, including type 2 diabetes mellitus, chronic kidney disease, heart failure, and secondary hyperparathyroidism.

## Case presentation

A 55-year-old male patient presented to our hospital with a chief complaint of cutaneous pruritus, along with symptoms of decompensated heart failure, including progressive worsening of baseline dyspnea and lower limb edema.

The patient reported an 8-month history of persistent, severe pruritus, worse at night, followed by the development of progressive, painless skin lesions that initially appeared on his upper limbs, subsequently involved lower limbs, sparing the trunk and face. He has a medical history of longstanding type 2 diabetes mellitus diagnosed 15 years ago, hypertension, and ischemic heart disease with a reduced ejection fraction of 25%. As well as chronic kidney disease (baseline serum creatinine 1.5–1.7 mg/dl) complicated with secondary hyperparathyroidism. His laboratory results showed an increase in serum creatinine (2.72 mg/dl), BUN (69.5 mg/dl), parathyroid hormone (119 pg/ml), 25-OH vitamin D (11.2 ng/ml), calcium (8.96 mg/dl), and phosphorus (5.11 mg/dl).

**Figure 1:**
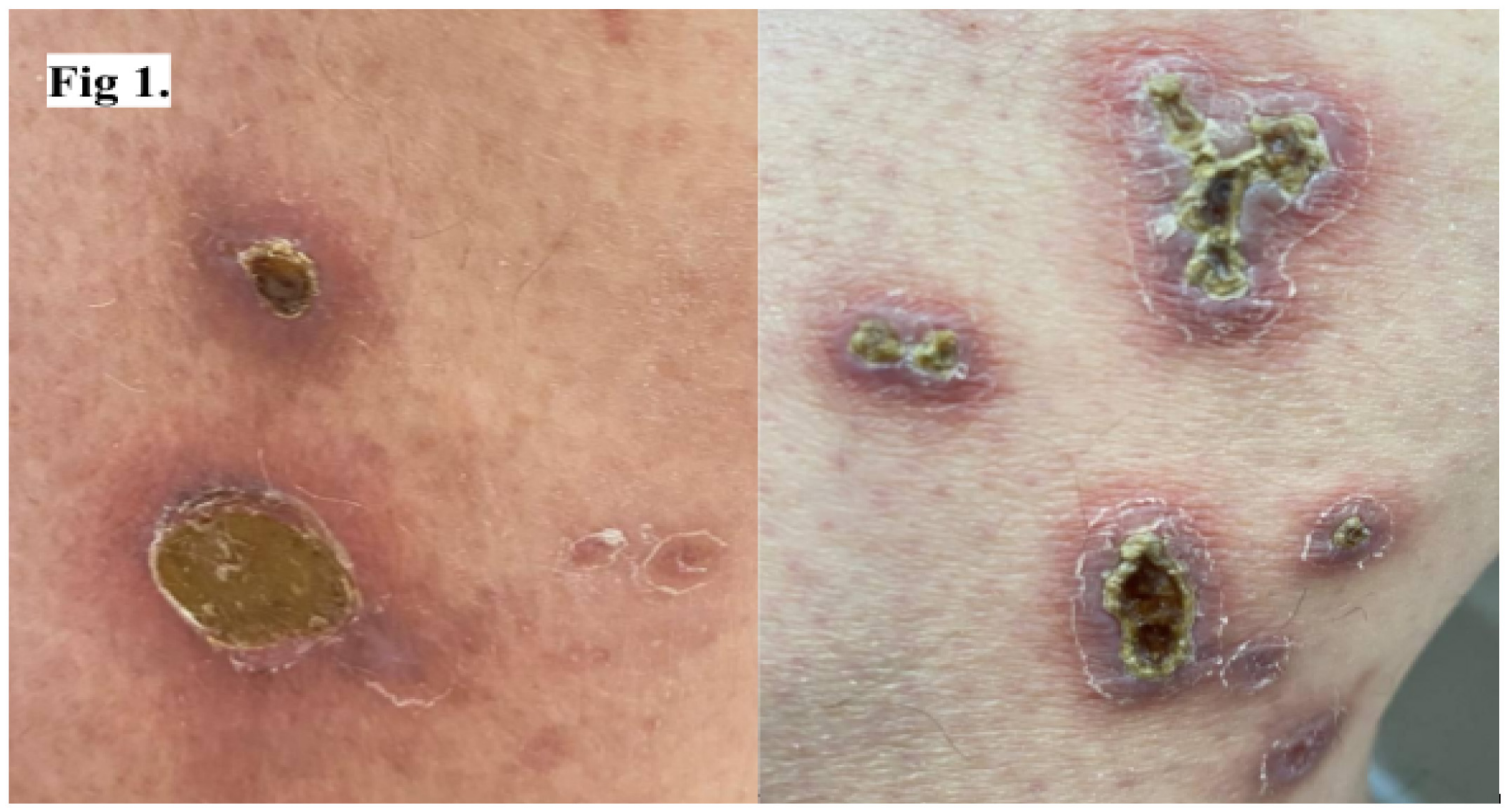
Multiple raised, firm, hyperkeratotic papules with central umbilication, erythematous borders, and adherent yellow-brown overlying crusts.

On examination, multiple diffuse, non-tender, well-demarcated keratotic papules measuring 10–15 mm in diameter were noted over the extensor aspects of both extremities. The lesions showed central umbilication, erythematous borders, and adherent yellow-brown crusts. The lesions were intensely pruritic, with secondary excoriations observed over several areas (Figure 1). In addition, the patient recognized the appearance of new lesions at the site of scratching, indicating a positive Koebner phenomenon.

The patient has no history of allergies, recent trauma, travel, insect bites, or change in medication, with no similar skin condition among family members. In addition, there were no symptoms suggesting rheumatologic diseases, including arthralgia, joint swelling, proximal muscle weakness, morning stiffness, photosensitivity, oral ulcers, dry and painful eyes, or Raynaud phenomenon. Serologic evaluation, including Anti-dsDNA (30.6 IU/ml), Anti-Jo 1 (4.2 IU/ml), Anti-scl 70 (6 IU/ml), RF (4.7 IU/ml), C4 (29 mg/dl), and C3 (105 mg/dl), was unremarkable. His initial labs, upon presentation, are reported in Table 1.

**Table 1:** Laboratory results at admission.

Lab	Result	Normal range
HGB	13.2^*^L g/dl	13.5–17.5 g/dl
MCV	69.8^*^L fl	80–97 fl
HbA1C	7%^*^H	<5.7%
TSH	4.41 μu/ml	0.4–4 μu/ml
Parathyroid H.	119^*^H pg/ml	9–80 pg/ml
Serum calcium	8.96 mg/dl	8.6–10.2 mg/dl
Phosphorus	5.11^*^H mg/dl	2.5–4.5 mg/dl
Bun	69.5^*^H mg/dl	6–20 mg/dl
Creatinine	2.72^*^H mg/dl	0.6–1.1 mg/dl
eGFR	24.4	>90 ml/min/1.73 m^2^
25-OH Vitamin-D	11.2 ng/ml	9–42 ng/ml
Anti-dsDNA	30.6 IU/ml	Negative (<30 IU/ml)
Anti-Jo 1	4.2 IU/ml	Negative (<20 IU/ml)
Anti-scl 70	6 IU/ml	Negative (<20 IU/ml)
RF	4.7 IU/ml	<14 IU/ml
C3	105 mg/dl	90–180 mg/dl
C4	29 mg/dl	10–40 mg/dl

**Figure 2:**
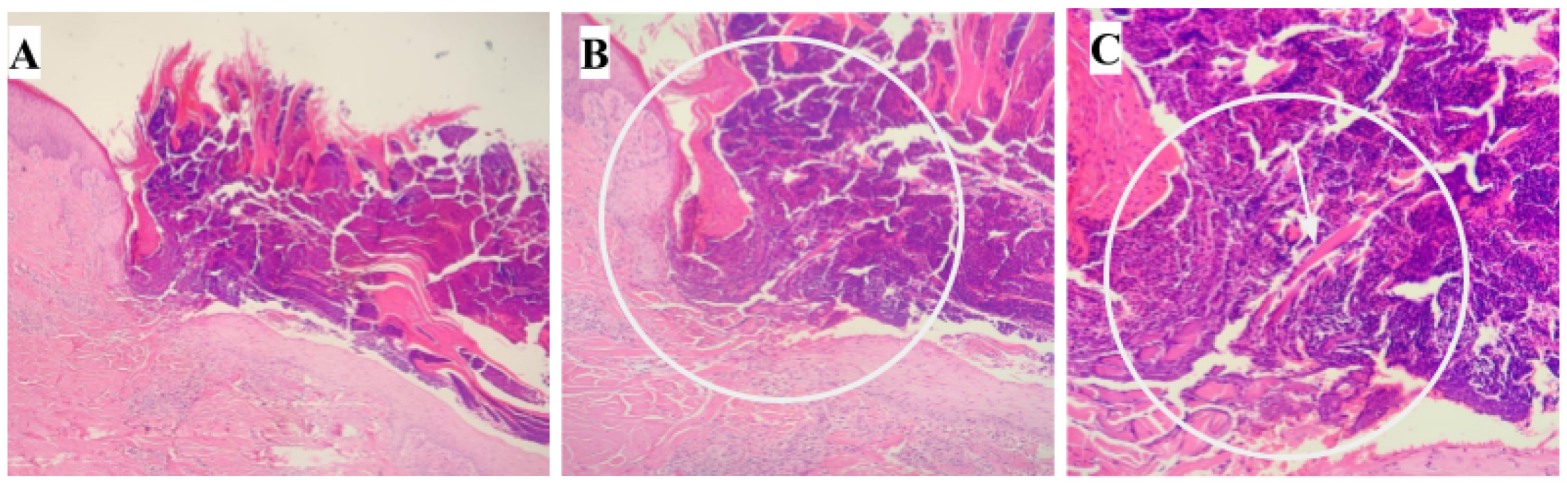
Histopathological findings of acquired reactive perforating collagenosis. **(A)** Low-power view demonstrating epidermal hyperkeratosis, mild spongiosis, acanthosis, and focal ulceration. **(B)** Intermediate-power view showing focal ulceration associated with aggregates of intracorneal neutrophils and a mild inflammatory infiltrate within the superficial dermis. **(C)** High-power view highlighting transepidermal elimination of altered collagen fibers, with the arrow indicating a vertically oriented collagen bundle perforating through the epidermis.

A punch skin biopsy with direct immunofluorescence evaluation was performed, demonstrating no evidence of immune complex deposition. Histopathological analysis demonstrated epidermal hyperkeratosis, mild spongiosis, acanthosis, and focal ulceration. The ulcerated area is associated with aggregates of intracorneal neutrophils with the presence of vertically oriented collagen bundles perforating through the epidermis. The superficial dermis showed patchy, mild, chronic inflammatory infiltrates with scattered neutrophils. There was no evidence of vasculitis or features suggestive of an immune-mediated bullous disorder. In conclusion, it was consistent with perforating collagenosis (Figure 2).

Initially, symptomatic treatment was applied using topical dexpanthenol and oral fexofenadine hydrochloride, which provided only minimal relief. Later on, it was replaced with topical corticosteroids, S.C omalizumab administered monthly, fexofenadine hydrochloride, and body moisturizer. Following up after 1 month revealed minimal improvement in pruritus; however, there was no change in skin lesion status.

## Discussion

Perforating dermatoses are a heterogeneous group of diseases characterized by the transepidermal elimination of dermal constituents, such as collagen, elastin, or fibrin (7). Classically, four distinct subtypes have been described based on clinical and histopathological features: Reactive Perforating Collagenosis (RPC), Elastosis Perforans Serpiginosa (EPS), Perforating Folliculitis, and Kyrle disease (7).

Reactive perforating collagenosis (RPC) is basically caused by the removal of damaged collagen fibers. The hereditary type generally presents with early onset during childhood or later in childhood at trauma sites, particularly in the arms and hands (8).

The acquired type was first described by Rapini et al. (9) in 1989. It typically occurs in adult patients with underlying comorbidities, most commonly during the fourth to fifth decades of life (10). ARPC is pre-dominantly observed in patients with chronic renal failure or diabetes mellitus, while associations with solid tumors, AIDS, lymphomas (3), liver disease, and hypothyroidism are less frequently reported (10).

While type 2 diabetes mellitus and chronic kidney disease are well-established comorbidities associated with ARPC, hyperparathyroidism has been reported less frequently in the literature and was also present in our patient (Table 2) (3, 11–13). The present case exhibited several characteristic features of ARPC, including severe pruritus, the Koebner phenomenon, characteristic papulonodular lesions, and histopathological evidence of transepidermal collagen elimination. Notably, the patient had multiple concomitant systemic conditions, including type 2 diabetes mellitus, chronic kidney disease, heart failure, and secondary hyperparathyroidism. The coexistence of these comorbidities may have contributed to both the development and persistence of the disease (3, 11–13).

**Table 2:** Summary of reported cases of reactive perforating collagenosis associated with hyperparathyroidism.

Profile	Symptoms	Duration before diagnosis	Lesion distribution	TEE material on biopsy	Associated disorders	Treatment	Outcomes
71 years old (3) male	Chronic pruritus	Not available	Arms and legs	Collagen fibers	Nephropathy Cardiomyopathy Elevated liver function Ascites Anemia HPT PVD COPD	Topical cleansing	Cleared in 6 weeks
73 years old (11) male	Chronic pruritus	10-months	Extremities and trunk	Collagen fibers	DM type 1 Retinopathy Cardiomyopathy Chronic renal failure Secondary HPT	Allopurinol 100 mg	Skin lesions and pruritus improved
54 years old (12) female	Recurrent rash with severe pruritus	5 years	Back and extensor surface of legs	Collagen and elastic fibers	CKD requiring hemodialysis HTN Hyperlipidemia HPT Post subtotal parathyroidectomy	Topical corticosteroids	The skin lesions improved
51 years old (12) male	Severe pruritus	1 month	Back	Collagen and elastic fibers	Hypertension DM Hyperlipidemia Hyperuricemia Coronary heart disease Hyperparathyroidism Diabetic retinopathy	Modifying the concentration and capacity of his peritoneal dialysis regimen	The dermatosis improved, lesions resolved, and itchiness was relieved
28 years old male (13)	Pruritus	Not available	Legs	Collagen and elastic fibers	CKD secondary to a Dent's disease Initiated CAPD in 2018 HPT secondary to CKD	Isotretinoin at a dosage of 0.5 mg/kg of body weight	Favorable subsequent evolution
44 years old male (13)	Pruritus	Not available	Legs	Collagen fibers	DM type 1 CKD secondary to DKD Initiated CAPD in April 2019 HPT secondary to CKD	Isotretinoin at a dosage of 0.5 mg/kg of body weight and heliotherapy	The patient responded well to the treatment, showing positive progress in their condition
58 years old male (13)	Pruritus	Not available	Back	Collagen fibers	DM type 2 Obesity Rheumatoid arthritis Hypothyroidism CKD secondary to DKD HPT secondary to CKD Started hemodialysis in April 2021	Corticosteroid therapy, isotretinoin at a dosage of 0.5 mg/kg of body weight, and phototherapy were initiated in an attempt to manage the condition	Despite interventions, the patient did not show optimal improvement in their condition

TEE, transepidermal elimination; HPT, hyperparathyroidism; PVD, peripheral vascular disease; COPD, chronic obstructive pulmonary disease; DM, diabetes mellitus; HTN, hypertension; CKD, chronic kidney disease; CAPD, continuous ambulatory peritoneal dialysis; DKD, diabetic kidney disease.

Consistent with the literature, pruritus and excoriation marks often precede the appearance of the papulonodular rash in many reported cases. Kurschat et al. (14) suggested that scratching secondary to pruritus may act as a primary trigger in the development of RPC. Clinically, ARPC lesions present as isolated papules with central keratotic plugs that may progressively enlarge from pinpoint size to 5 to 6 mm in diameter, exhibiting a central umbilication (15).

ARPC diagnosis is based on clinical assessment supported by histopathological evidence, in accordance with the diagnostic criteria proposed by Faver et al. (3) which require the presence of umbilicated papules or nodules with a central keratotic plug in an adult patient (age >18 years) and histopathological evidence of transepidermal elimination of dermal connective tissue material. In our case, keratotic plugs and transepidermal perforation were observed, with Masson's trichrome stain (MTS) confirming collagen perforation, meeting the diagnostic criteria.

At the histological level, features of ARPC generally include altered collagen bundles in the initial phase, followed by a cup-shaped epidermal invagination filled with keratinized material (15). While EVG staining visualizes vertical collagen fibers in red, MTS identifies collagen in blue-green coloration (4).

The pathogenesis of RPC remains incompletely understood. Mehregan first proposed that superficial trauma may contribute to its development (1). In response to injury, structural alterations may occur, resulting in increased hematoxylin affinity in the connective tissue of the papillary dermis or the walls of superficial capillaries. These changes are followed by epidermal atrophy and parakeratosis, with transepidermal elimination of collagen (1).

Several studies also reveal a possible correlation between diabetic microangiopathy and ARPC (16–18). Dermal hypoxia may promote the separation of collagen fibers and the destruction of inter-keratinocyte linkages. Therefore, diabetic microvasculopathy and the resulting hypoxic state may pre-dispose to the development of ARPC (19).

An additional theory proposed by Akoglu et al. (16) suggests elevated expression of advanced glycation end products (AGEs) and their multiligand transmembrane receptor (RAGE) in the microvascular endothelium, fibroblasts, and inflammatory cells, which may provide insight into the correlation between ARPC and systemic diseases, including CKD, DM, and atherosclerotic diseases.

In diabetic patients, scratching exposes the basal keratinocytes (KC) of the damaged endothelial basement membrane (BM) to AGEs. Interaction with CD36 of RAGE promotes keratinocyte terminal differentiation, followed by upward migration with deposition of AGE-modified collagen into the epidermis. Moreover, exposure of KCs to these altered proteins further damages the BM through upregulating matrix metalloproteinase 9 (MMP-9), a potent enzyme involved in the degradation of extracellular matrix (ECM) proteins (20).

Treatment approaches of RPC are largely based on limited evidence and case reports. Management primarily focused on relieving pruritus to prevent disease exacerbation and the presence of new lesions secondary to Koebnerization. Therapeutic regimens include topical or intralesional corticosteroids as well as topical or systemic retinoids. Phototherapy, such as narrow-band or broad-band ultraviolet B (UVB) or psoralen plus UVA (PUVA), may also be beneficial, especially for pruritus (21). Additional treatment options described in the literature include antibiotics such as doxycycline, along with procedural interventions including cryotherapy, surgical debridement, and laser therapy (2).

In the present case, treatment with topical corticosteroids, antihistamines, moisturizers, and omalizumab resulted in only minimal improvement in pruritus after 1 month, while the skin lesions remained unchanged. The limited response may be related to the chronic nature of the disease, the presence of multiple systemic comorbidities, and the relatively short follow-up period. Clinical outcomes reported in the literature have been variable, with some patients achieving significant clinical improvement, whereas others demonstrate persistent or refractory disease despite multimodal therapeutic interventions (14–21).

Oral allopurinol may be considered a favorable therapeutic option for patients with ARPC-associated end-stage renal disease (ESRD) based on its mechanism of action. Specifically, inhibition of xanthine oxidase leads to decreased production of reactive oxygen species, which consequently attenuates the formation of AGE and the subsequent activation of KC via the CD36 receptor (11).

Promising future therapeutic drugs may focus on the AGE–RAGE axis, such as aminoguanidine, which inhibits the production of AGE and may act as a potential inhibitor of this pathway in ARPC, particularly when the perforating process is a consequence of diabetes (22).

## Conclusion

Reactive perforating collagenosis is a rare dermatological disorder marked by the transepidermal expulsion of modified collagen fibers. The acquired type pre-dominantly manifests in adults with pre-existing systemic conditions, most commonly diabetes mellitus and chronic renal insufficiency. Nonetheless, its association with hyperparathyroidism appears infrequent and has been minimally documented in the literature. Diagnosis is confirmed with histopathology and clinical presentation. The precise pathophysiology is uncertain; however, elements like trauma, scratching, and microvascular alterations may play a role in disease progression. Effective management focuses on controlling pruritus, preventing new lesion development, and addressing underlying comorbidities. The present case further highlights the uncommon association of ARPC with secondary hyperparathyroidism and illustrates the therapeutic challenges that may arise in patients with multiple concurrent systemic diseases. Additional research is necessary to enhance comprehension of the pathogenesis and to establish standardized treatment protocols that will improve the quality of life for affected patients.

## Data Availability

The raw data supporting the conclusions of this article will be made available by the authors, without undue reservation.
